# The Effects of *Lactobacillus casei* on Glycemic Response, Serum Sirtuin1 and Fetuin-A Levels in Patients with Type 2 Diabetes Mellitus: A Randomized Controlled Trial

**DOI:** 10.29252/.23.1.68

**Published:** 2019-01

**Authors:** Leila Khalili, Beitullah Alipour, Mohammad Asghari Jafar-Abadi, Ismail Faraji, Tohid Hassanalilou, Mehran Mesgari Abbasi, Elnaz Vaghef-Mehrabany, Mahmood Alizadeh Sani

**Affiliations:** 1 Department of Nutrition, Faculty of Nutrition and Food Sciences, Tabriz University of Medical Sciences, Tabriz, Iran; 2 Department of Community Nutrition, Faculty of nutrition and food sciences, Tabriz University of Medical Sciences, Tabriz, Iran; 3 Department of statistics and Epidemiology, Faculty of Health, Tabriz University of Medical Sciences, Tabriz, Iran; 4 Internist, fellow of Endocrinology, Tabriz University of Medical Sciences, Tabriz, Iran; 5 Drug Applied Research Center, Tabriz University of Medical Sciences, Tabriz, Iran; 6 Department of Nutrition, Biochemistry and Diet Therapy, Faculty of nutrition and food science, Tabriz University of Medical Sciences, Tabriz, Iran; 7 Department of Food Science and Technology, Faculty of nutrition and food sciences, Tabriz University of Medical Sciences, Tabriz, Iran

**Keywords:** *Lactobacillus casei*, Probiotics, Type 2 diabetes mellitus

## Abstract

**Background:**

Type 2 diabetes mellitus (T2DM) is related to the gut microbiota with numerous molecular mechanisms. Modulating the gut microbiota by probiotics could be effective in management of T2DM. The aim of the present trial was to evaluate the effect of *Lactobacillus casei* on glycemic control and serum sirtuin1 (SIRT1) and fetuin-A in patients with T2DM.

**Methods:**

Forty patients with T2DM (n = 20 for each group) were divided into intervention (probiotic) and placebo groups. The intervention group received a daily capsule containing 10^8^ cfu of *L. casei* for eight weeks. The patients in placebo group took capsules containing maltodextrin for the same time duration. Anthropometric measurements, dietary intake questionnaires, and blood samples were collected, and the patients were assessed by an endocrinologist at the beginning and at the end of the trial.

**Results:**

Fasting blood sugar, insulin concentration, and insulin resistance significantly decreased in probiotic group compared with placebo group (-28.32 [-50.23 to -6.41], 0.013; -3.12 [-5.90 to -0.35], 0.028; -32.31 [-55.09 to -9.54], 0.007, respectively). Moreover, HbA1c reduced after intervention, but the reduction was not significant (-0.45 [-0.96 to 0.05], 0.077). In comparison with placebo, the *L. casei* supplementation significantly increased SIRT1 and decreased fetuin-A levels at the end of the trial (0.52 [0.026 to 1.02], 0.040; -17.56 [-32.54 to -2.58], 0.023, respectively).

**Conclusion:**

*L. casei* supplementation affected SIRT1 and fetuin-A levels in a way that improved glycemic response in subjects with T2DM. Affecting the SIRT1 and fetuin-A levels introduces a new known mechanism of probiotic action in diabetes management.

## INTRODUCTION

Type 2 diabetes mellitus (T2DM), a metabolic disorder known by high blood glucose, is caused by the combination of not-sufficient secretion of insulin and insulin resistance[1,2]. Diabetes is among the top ten causes of death globally. This metabolic illness along with three other diseases (cardiovascular diseases, cancer, and respiratory diseases) accounts for over 80% of all premature non-communicable diseases deaths.

According to a recent report by International Diabetes Federation, 425 million adults suffer from diabetes, and 1 in 2 remains undiagnosed. The global prevalence of diabetes in adults of 20-79 years is now 7.3% (4.8-11.9%) that is estimated to reach 8.3% (5.6%-13.9%) by 2045[3]. Evidence has shown that the intestinal microbiota could affect the host by influencing bile acid metabolism, body weight, pro-inflammatory status, and insulin resistance, as well as modulation of gut hormones. Moderating gut microbiota by the use of probiotics, prebiotics, and antibiotics could have beneficial effects on glucose metabolism and insulin resistance[4]. Probiotics, the live microorganisms, present health benefits to the host, especially when administered in sufficient amounts[5,6]. Physiologic functions of probiotics would lead to the modulation of gut microbiota and can affect appetite, food intake, body weight, and body metabolic functions through gastrointestinal pathways[7].

Sirtuin1 (SIRT1), a nicotinamide adenine dinucleotide-dependent deacetylase, is a principal modulator of energy metabolism and exerts positive impacts on glucose homeostasis and insulin sensitivity. SIRT1 activators improve whole-body glucose homeostasis and insulin sensitivity in adipose tissue, skeletal muscle, and liver. Evidence has revealed that the endogenous activators of SIRT1 increase after calorie restriction and weight loss[8].

Fetuin-A, a serum protein, is expressed and secreted by adipocytes and hepatocytes, and its level is up-regulated in hepatic steatosis and other metabolic disorders[9,10]. By binding the insulin receptor and inhibiting auto-phosphorylation of tyrosine kinase, fetuin-A could decrease insulin signaling in cell culture models[11]. It has also been shown that fetuin-A concentration decreases by weight loss, indicating the reversibility of the increased fetuin-A levels[12]. Thus, increasing the SIRT1 level and decreasing the fetuin-A level could be promising new therapeutic approaches for treating metabolic disorders such as T2DM. Considering the potential of probiotic bacteria, the aim of the present trial was to investigate the effects of *Lactobacillus casei* supplementation on the glycemic response and SIRT1 and fetuin-A levels in patients with T2DM.

## MATERIALS AND METHODS

### Subjects

An 8-week, parallel-group, randomized controlled trial was conducted in the Sheykholrayis Polyclinic of Tabriz University of Medical Sciences, Tabriz, Iran. The recruitment process of participants began in September 2016, and the intervention was carried out in January 2017.

The target population of the present study was patients with T2DM. Subjects were contacted a day before commencing the supplementation, and the study was thoroughly explained to them. Volunteers were composed of 44 patients with T2DM, 30-50 years of age, and body mass index (BMI) lower than 35 kg/m^2^. All patients had been diagnosed with T2DM for at least one year. Exclusion criteria were smoking, the presence of kidney, liver, and/or inflammatory intestinal disease, thyroid disorders, immunodeficiency diseases, required insulin injections, use of nutritional supplements within the previous three weeks of testing, use of estrogen or progesterone, pregnancy or breast-feeding, consuming any type of antibiotics, and consuming any other probiotic products within the previous two months of testing. Primary endpoints were the promotion of SIRT1, reduction of fetuin-A levels, and control of glycemic response, and secondary endpoint was the management of dietary intake and body weight.

The sample size for the current study was calculated on the basis of FBS results reported by Ostadrahimi *et al*.[13] with a confidence level of 95% and a power of 80%, which was found to be 18 patients. Taking into account the probable dropout of patients during the intervention course as well as the patients who may not adhere to the study protocol, 22 patients with T2DM were recruited for each group.

### Study design and measurements

Of 44 patients who had met the inclusion criteria, 4 were excluded because of their unwillingness to participate in the study (Fig. 1). Subjects were randomly assigned to the probiotic (n = 20) and placebo (n = 20) groups, using a block randomization procedure with stratified subjects in each block based on sex and age. The allocation of the intervention or control group was concealed from the researchers, and the probiotic and placebo capsules had both an identical appearance and labeled information. Therefore, neither the subjects nor the investigators were aware of the treatment assignments in this double-blinded study. Over eight weeks, both groups consumed probiotic capsules containing 10^8^ cfu *L. casei* or placebo capsules. Considering the buffering capacity of the food on the survival of probiotic microbes during gastrointestinal transit[14], the patients were asked to take the capsules with or just prior to a meal containing some fats. All patients were asked, throughout the 8-week trial, to maintain their usual dietary habits and lifestyle. The patients were instructed to keep the capsules under refrigeration and to avoid any changes in medication, if possible.

Arrangements were made so that the patients would receive the eight-week supply of their probiotic or placebo capsules at the beginning of the trial and were asked to take a capsule daily. Compliance with the capsule consumption guidelines was monitored by telephone interviews once a week. Information on demographic and anthropometric measurements and fasting blood samples were collected at the beginning and at the end of the trial. Nutrient intakes during three days were estimated using a 24-h dietary recall at the beginning, in the middle, and at the end of the study. Three-day averages of total energy intake (TEI) and macro-nutrients intake were analyzed by Nutritionist 4 software (First Databank, Hearst Corp, San Bruno, CA, USA). International Physical Activity Questionnaire (IPAQ)[15] was completed for participants to assess physical activity level.

Anthropometric measurements were recorded by trained personnel. A blood sample was drawn from each patient after an overnight fasting. All whole blood and serum samples were collected and kept at -70°C until assay. Blood samples were analyzed at the Drug Applied Research Center (Tabriz University of Medical Sciences, Tabriz, Iran).

Fasting blood glucose was measured using the standard enzymatic method with the Pars Azmun kit (Karaj, Iran). Glycated hemoglobin (HbA1c) in the whole blood was measured by cation exchange chromatography with the NycoCard HbA1c kit (Oslo, Norway). Insulin concentration was determined by a chemiluminescent immunoassay using a LIAISON analyzer (Diameter, Italy). To measure insulin resistance, we used insulin resistance index, HOMA-IR (homeostatic model assessment of insulin resistance), based on the following formula: HOMA1-IR = FPI (mg/dl) × FPG (mg/dl))/22.5. Serum fetuin-A and SIRT1 concentrations were measured by human ELISA kits (Diameter, Italy and Bioassay Technology Laboratory, China).

The present study was conducted in accordance with the guidelines laid down in the Declaration of Helsinki, and all procedures were approved by the Ethics Committee of Tabriz University of Medical Sciences (no. IR.TBZMED.REC.1395.402). A written informed consent was obtained from each patient.

### Intervention procedure

Hard yellow gelatin capsules were used as delivery vehicle in the present study. *L. casei* (Chr. Hansen, Denmark) was the active agent of the probiotic capsules, and maltodextrin was used as the excipient. The capsules were prepared using a capsule filling device under aseptic condition. To check the quality of probiotic capsules and ensure that an adequate dose of the probiotic was consumed by the experiment group (at least 10^8^ CFU/day), a food technologist checked the bacterial count of the capsules at the baseline, in the middle and at the end of the trial period, by culturing the contents of three capsules at each. The capsules were cultured with the use of MRS agar (De Man, Rogosa, and Sharpe agar) via serial dilution and the pour plate technique. Bacterial enumeration of the capsules showed that the capsules were composed of a minimum of 10^8^ colony-forming units of *L. casei* during the study period. The placebo capsules contained only maltodextrin. Since the bacterial count of the excipient could confound the outcomes of the study, the powder was cultured to ensure it was free of pathogens. Capsule count was performed by the researcher at the end of the study to evaluate compliance.

### Statistical analyses

Statistical analysis was performed by SPSS software (ver. 17; SPSS Inc. IL, Chicago, USA). Normality of the numeric variables was checked by Kolmogorov-Smirnov test[16]. Data were presented using mean (SD), median (min-max) for the numeric normal, and non-normal variables, respectively, as well as by the percentage of frequency for categorical variables. The between-group comparisons of baseline measures and demographic variables were conducted with independent *t*-test and/or Chi-square test where appropriate. For within-group comparisons, paired *t*-tests were used, where before and after intervention measurements were taken. To assess the effect of intervention, the analysis of covariance (ANCOVA) was used to control baseline measurements and confounders. In all analyses, *p* values less than 0.05 were considered statistically as significant.

## RESULTS

Forty patients with T2DM were recruited in the present clinical trial (n = 20 for each group). Capsule bacterial counts showed a good compliance in those precipitants that completed the study, and no adverse effects were reported. There were no significant differences between the two groups with regard to any of the baseline characteristics (Table 1), biomedical parameters (Table 2), anthropometric measurements (Table 3), and dietary intake (Table 4) at the beginning of the study.

**Table 1:** Characteristics and medication intake of the participants under study

Variable	Placebo group (n = 20)	Intervention group (n = 20)	*p* value
Age (Year)*	45.00 (5.37)	43.95 (8.14)	0.629
Diabetes Duration (Month)*	3.67 (4.00)	4.00 (3.81)	0.794
Sex**			
Male	7 (35.0)	7 (35.0)	1.00
Female	13 (65.0)	13 (65.0)	
Marital status**			
Single	2 (10.0)	2 (10.0)	1.00
Married	18 (90.0)	18 (90.0)	
Education**			
Primary	0 (0.0)	1 (5.0)	1.00
Under diploma	10 (50.0)	6 (30.0)	
Diploma	5 (25.0)	11 (50.0)	
Higher education	5 (25.0)	2 (10.0)	
Physical activity**			
Non-active	10 (50.0)	5 (25.0)	0.247
Light-active	8 (40.0)	11 (55.0)	
Active	1 (5.0)	3 (15.0)	
Heavy-active	1 (5.0)	1 (5.0)	
Diet Therapy (year)**			
Yes	9 (45.0)	8 (40.0)	0.749
No	11 (55.0)	12 (60.0)	
Glibenclamide**			
Not used	13 (65.0)	12 (60.0)	0.744
Using more than one/d	7 (35.0)	8 (40.0)	
Metformin**			
1-2 tablet/d	9 (45.0)	8 (40.0)	0.749
3-4 tablet/d	11 (55.0)	12 (60.0)	

* Data are expressed as mean (SD) and *p* value based on independent *t*-test;

** Frequency (percent) is reported and *p* value based on Chi-squared test.

**Table 2:** The Effect of *L. casei* supplementation on biochemical parameters

Variable	Placebo group (n = 20)	Intervention group (n = 20)	MD (95%CI)	*P* value
FBS (mg/dl)				
Before	149.40 (36.86)	164.20 (46.91)	14.79 (-12.21 to 41.80)	0.274*
After	150.59 (36.72)	135.84 (43.01)	-28.32 (-50.23 to -6.41)	0.013**
MD (95% CI), *p* value	1.18 (-5.46 to 7.83), 0.71	-28.35 (-45.39 to -11.31), 0.002		
HbA1c (%)				
Before	6.83 (0.95)	7.30 (0.65)	0.46 (-0.05 to 0.98)	0.081*
After	7.13 (0.99)	6.84 (0.99)	-0.45 (-0.96 to 0.05)	0.077**
MD (95% CI), *p* value	0.08 (-0.10 to 0.26), 0.379	-0.24 (-0.60 to 0.12), 0.190		
Insulin (mU/mL)				
Before	17.89 (6.77)	17.22 (7.21)	-0.67 (-5.15 to 3.81)	0.764*
After	18.31 (7.48)	14.89 (6.01)	-3.12 (-5.90 to -0.35)	0.028**
MD (95% CI), *p* value	0.41 (-0.28 to 1.11), 0.229	-2.33 (-4.48 to -0.18), 0.035		
HOMA.IR				
Before	117.03 (46.84)	123.14 (44.82)	6.11 (-23.23 to 35.46)	0.676*
After	121.59 (54.67)	93.42 (45.34)	-32.31 (-55.09 to -9.54)	0.007**
MD (95% CI), *p* value	4.56 (-2.29 to 11.43), 0.180	-29.72 (-45.62 to -13.82), 0.001		
SIRT1 (ng/mL)				
Before	5.87 (2.17)	6.04 (1.51)	0.16 (-1.03 to 1.36)	0.262*
After	5.83 (2.75)	6.96 (2.12)	0.52 (0.026 to 1.02)	0.040**
MD (95% CI), *p* value	-0.04 (-0.51 to 0.43), 0.862	0.52 (0.17 to 0.87), 0.006		
Fetuin-A (µg/ml)				
Before	139.95 (45.36)	119.53 (46.95)	-20.41 (-49.97 to 9.14)	0.170*
After	145.00 (45.86)	107.63 (41.79)	-17.56 (-32.54 to -2.58)	0.023**
MD (95% CI) *p* value	5.05 (-2.59 to 12.69), 0.183	-11.90 (-20.29 to -3.51), 0.008		

MD, mean difference; CI, confidence interval; FBS, fasting blood sugar; HbA1c, glycated hemoglobin; HOMA-IR, homeostatic model assessment of insulin resistance; SIRT1, Sirtuin1. Data are expressed as mean (SD).

* For baseline between group comparisons, *p* values are based on independent *t*-test;

** After intervention between group comparisons, *p* values and CI are based on analysis of covariance adjusted for baseline measures, BMI, energy intake, and drug intake. For within group comparisons, *p* values and confidence intervals are based on paired *t*-test.

**Table 3:** The Effect of *L. casei* supplementation on anthropometric variables and blood pressure

Variable	placebo group (n = 20)	Intervention group (n = 20)	MD (95%CI)	*P* value
Weight (Kg)				
Before	83.45 (15.84)	77.15 (13.58)	-6.30 (-15.74 to 3.14)	0.185
After	83.70 (15.49)	75.95 (13.70)	-1.52 (-2.31 to -0.76)	<0.001
MD (95% CI), *p* value	0.25 (-0.25 to 0.75), 0.309	-1.20 (-1.81 to -0.58), 0.001		
Waist circumference (cm)				
Before	102.90 (10.22)	97.50 (8.94)	-5.40 (-11.54 to 0.74)	0.083
After	102.15 (9.9)	95.35 (8.49)	-1.77 (-3.36 to -0.18)	0.029
MD (95% CI), *p* value	-0.75 (-1.87 to 0.37), 0.179	-2.15 (3.30 to -0.99), 0.001		
WHR				
Before	0.95 (0.05)	0.94 (0.08)	-0.01 (-0.05 to 0.03)	0.647
After	0.94 (0.06)	0.92 (0.07)	-0.01 (-0.02 to 0.00)	0.052
MD(95% CI), *p* value	-0.006 (-0.015 to 0.003), 0.18	-0.020 (-0.031 to -0.009), 0.001		
BMI (kg/m^2^)				
Before	31.94 (5.76)	29.50 (3.34)	-2.43 (-5.47 to 0.60)	0.113
After	32.19 (5.49)	29.02 (3.36)	-0.84 (-1.26 to -0.41)	<0.001
MD(95% CI), *p* value	0.249 (-0.11 to 0.61), 0.168	-0.485 (-0.73 to -0.23), 0.001		
SBP (mmHg)				
Before	114.50 (5.10)	114.00 (5.02)	-0.50 (-3.74 to 2.74)	0.757
After	115.00 (5.12)	109.05 (23.60)	-5.27 (-15.43 to 4.89)	0.30
MD (95% CI), *p* value	0.50 (-0.54 to 1.54), 0.330	-4.95 (-15.31 to 5.41), 0.330		
DBP (mmHg)				
Before	66.50 (4.89)	70.00 (6.48)	3.50 (-0.17 to 7.17)	0.062
After	68.00 (6.15)	67.00 (4.70)	-1.48 (-3.97 to 1.00)	0.235
MD (95% CI), *p* value	0.50 (-0.54 to 1.54), 0.330	-2.00 (-4.44 to 0.44), 0.104		

MD, mean difference; CI, confidence interval; WHR, waist to heap ratio; BMI, body mass index; SBP, systolic blood pressure; DBP, diastolic blood pressure. Data are expressed as mean (SD). For baseline between group comparisons, *p* values are based on independent *t*-test. For after intervention between group comparisons, *p* values and confidence intervals are based on analysis of covariance adjusted for baseline measures. For within group comparisons, *p* values and confidence intervals are based on paired *t*-test.

The effect of *L. casei* supplementation on biochemical parameters is shown in Table 2. After the two-month intervention, FBS, serum insulin level, and HOMA.IR index significantly reduced in the intervention group (*p* = 0.002, *p* = 0.035, and *p* = 0.001, respectively). Moreover, the between-group differences for the mentioned glycemic response parameters were significant (*p* = 0.013, *p* = 0.028, and *p* = 0.007, respectively). Evaluation of HbA1c after two-month supplementation showed no significant reduction in probiotic group.

As presented in the Table 2, the level of SIRT1 significantly increased after two-month intervention in probiotic group (*p* = 0.006). Moreover, a significant decrease in the serum fetuin-A level was found in the probiotic group (*p* = 0.008). The between-group changes were statistically significant (*p* = 0.040 and *p* = 0.023, respectively).

The effect of consumption of *L. casei* on anthropometric variables is shown in Table 3. Consumption of *L. casei* for two months significantly decreased weight, BMI, and waist circumference in probiotic group compared with placebo group (*p* < 0.001, *p* < 0.001, and *p* = 0.029, respectively). Moreover, the within-group changes for the three parameters were significant in probiotic group (*p* < 0.001, *p* = 0.001, and *p* = 0.001, respectively). Although the between-group change for waist to hip ratio was not significant, the within-group change was statistically significant (*p* = 0.001).

The analysis of dietary questionnaires, shown in Table 4, revealed that TEI and the intake of macronutrients were significantly changed after probiotic supplementation. As indicated in Table 4, TEI and the intake of carbohydrate, fat, and protein significantly reduced during the intervention period in the intervention group (*p* = 0.003, *p* < 0.001, *p* < 0.001, and *p* = 0.001, respectively). The between-group changes for TEI and protein were significant in 3^rd^ evaluation (*p* = 0.001 and *p* < 0.001, respectively); moreover, the between-group changes for carbohydrate and fat intake were significant in both 2^nd^ (*p* < 0.001 and *p* = 0.003, respectively) and 3^rd^ (*p* = 0.002 and *p* = 0.009, respectively) evaluation.

**Table 4:** The effect of *L. casei* supplementation on dietary intake

Variable	Placebo group (n = 20)	Intervention group (n = 20)	MD (95%CI)	*p* value
Total energy intake (Cal)				
Before	2131.62 (368.83)	2076.81 (394.95)	-54.32 (-298.83 to 190.29)	0.656
Mid	2165.28 (352.19)	1954.62 (405.71)	-33.71 (-68.48 to 1.05)	0.057
After	2147.85 (387.22)	1853.94 (398.17)	-35.80 (-55.47 to -16.13)	0.001
*p* value	0.080	0.003		
CHO (g)				
Before	295.02 (48.69)	284.87 (45.53)	-10.15 (-43.24 to 22.94)	0.387
Mid	297.79 (48.42)	282.00 (53.68)	-5.76 (-7.81 to -3.72)	<0.001
After	295.95 (52.50)	277.12 (52.15)	-8.67 (-13.82 to -3.52)	0.002
*p* value	.310	<.001		
Protein (g)				
Before	79.91 (13.83)	77.68 (14.87)	-2.22 (-11.41 to 6.96)	0.627
Mid	81.21 (13.20)	77.04 (15.21)	-1.08 (-2.33 to 0.16)	0.087
After	80.71 (14.31)	75.89 (14.93)	-1.35 (-1.89 to -0.81)	<0.001
*p* value	0.066	0.001		
Fat (g)				
Before	71.03 (12.29)	69.00 (13.23)	-2.02 (-10.20 to 6.15)	0.619
Mid	72.18 (11.73)	68.57 (13.25)	-1.64 (-2.68 to -.60)	0.003
After	73.25 (14.07)	66.94 (13.10)	-4.29 (-7.45to -1.13)	0.009
*p* value	0.248	<0.001		

MD, mean difference; CI, confidence interval; CHO, carbohydrate. Data are expressed as Mean (SD). For baseline between group comparisons, *p* values are based on independent *t*-test. For mid and after intervention between group comparisons, *p* values and confidence intervals are based on analysis of covariance adjusted for baseline measures. For within group comparisons, *p* values are based on repeated measure analysis of variance and Sidak post hoc tests.

## DISCUSSION

Controlling diabetes by natural food without side effects is a challenge for medical nutrition therapy of diabetes. This is the first study evaluating the effect of *L. casei* supplementation on serum SIRT1 and fetuin-A in patients with T2DM.

The outcomes of the present trial showed that eight weeks of *L. casei* supplementation improved glycemic response and increased SIRT1 and decreased fetuin-A level compared with placebo group. In comparison with placebo group, *L. casei* supplementation decreased the intake of total energy and macronutrients significantly; moreover, it improved anthropometric parameters in probiotic group.

Improvements in glycemic control by probiotic bacteria, as seen in this study, were in accordance with other similar studies conducted previously[17-20]. The antidiabetic property of *Bifidobacteria* and *Lactobacillus* has been evaluated in human and animal studies[19,21-23]. Previous studies have shown that probiotic treatment could reduce blood glucose concentration in diabetic status[21,24]. Ejtahed *et al*.[2] have declared that probiotic yogurt, containing *Lactobacillus acidophilus* and *Bifidobacterium lactis*, decreased FBS and HbA1c in patients with T2DM. Ostadrahimi *et al*.[13] have shown that probiotic fermented milk, containing *L. acidophilus*, *L. casei*, and *Bifidobacteria* decreased fasting blood glucose and HbA1c compared with control group.

Several possible mechanisms of hypoglycemic effect of probiotics are discussed. Probiotics can affect gut bacteria to produce insulin-tropic polypeptides and GLP-1 (glucagon-like peptide-l), thus increasing glucose uptake by muscle and stimulating the liver absorption of blood glucose[25]. The immune-modulatory and anti-inflammatory effects of probiotics and the modulation of intestinal microbiota composition are other possible mechanisms[2].

HbA1c reduced after 2-month consumption of probiotic capsules; however, the reduction was not significant. Some of the similar studies have indicated that probiotics could reduce HbA1c in patients with T2DM[2,13]. However in a meta-analysis of randomized, controlled trials, Li *et al*.[26] have reported that there is no significant differences in HbA1c between the treatment and the control groups. The present study and most of the similar trials, evaluating the effect of probiotics on glycemic modulation in patients with T2DM, last for less than 3 months. A three-month period is needed to find the complete effect of the intervention on HbA1c.

The improved fasting insulin level found in this trial was in accord with the study conducted by Firouzi *et al*.[27] in which multi-strain probiotics significantly decreased the fasting insulin level in probiotics group after a 12-week intervention. The results of a meta-analysis, conducted by Yao *et al*.[28], demonstrated that probiotics supplementation was associated with a significant reduction in fasting insulin level in patients with T2DM.

After eight-week intervention, the insulin resistance improved in probiotic group. The result was in accordance with other similar investigations[29,30]. Andreasen *et al*.[19] have revealed that consuming *L. acidophilus* NCFM for four weeks improved insulin sensitivity in comparison with placebo. Similarly, Kobyliak *et al*.[31] have found that supplementation with multi-probiotic for eight weeks is associated with a significant reduction of HOMA-IR. By reducing the oxidative stress and inflammatory response in diabetes, probiotics can improve insulin resistance[32]. Specific probiotic strains improve the function of intestinal barrier[33] and reduce the translocation of microorganisms and their derivatives[34] to the systemic circulation, so reducing the pro-inflammatory cytokines production[35]. Studies have shown that special strains of lactic acid bacteria have antioxidant properties[36,37]. The anti-oxidative mechanisms of probiotics could be assigned to reactive oxygen species scavenging, enzyme inhibition, metal ion chelation, and ascorbate autoxidation inhibition[37]. Therefore, it can be understood that by improving inflammation and oxidative stress, probiotics can improve insulin resistance in subjects with T2DM.

As the results shown in Table 1, the levels of SIRT1 and Fetuin-A were significantly affected by probiotic consumption. The effect of probiotic supplementation on SIRT1 and/or fetuin-A was not evaluated in previous studies. Sirtuins, ubiquitous deacetylases, are metabolism regulators and energy homeostasis[38]. SIRT1 has a positive impact on metabolic disorders, including obesity, liver steatosis, and diabetes mellitus[39]. Due to its deacetylation activity, SIRT1 influences many steps of glucose metabolism in liver, pancreas, muscle, and adipose tissue and regulates insulin secretion[40,41]. It has been demonstrated that the SIRT1 expression in the adipose tissue of lean subjects is higher than that in obese individuals. According to previous investigations, it seems that weight loss associates with blood SIRT1 level changes. Mariani *et al*.[38] have found that there is a link between the reduction of body fat mass and increased plasma SIRT1; moreover, they showed that, in addition to the tissue levels, the circulating SIRT1 could be increased by a negative caloric balance. Calorie restriction has been reported to increase SIRT1 protein levels and activity in mice, rats, and humans. Moreover, it has been reported that SIRT1 gene variants are related to energy expenditure[42,43].

Fetuin-A, a circulating glycoprotein that is secreted by the liver and adipose tissues, inhibits insulin receptor tyrosine kinase activity in animal studies[44]. Fetuin-A knockout mice have enhanced glucose sensitivity, resistance to weight gain, and lower serum-free fatty acid levels[45]. In humans, the liver-secreted fetuin-A is associated with atherosclerosis, insulin resistance, T2DM, and metabolic syndrome[46]. In a cross-sectional analysis, Ismail *et al*.[12] have revealed that fetuin-A levels were higher in children and adults with obesity and metabolic syndrome. They reported that weight loss can reduce fetuin-A level. Haukeland *et al*.[47] have demonstrated that substantial weight loss in 21 children leads to a significant decrease in fetuin-A concentrations. Brix *et al*.[48] have reported that elevated fetuin-A levels in morbid obesity decreases after bariatric surgery. Additionally, caloric restriction is another factor that can decrease both fetuin-A levels and insulin resistance, as stated by Choi *et al*.[46]. They have found that caloric restriction significantly decreases expression and circulating levels of fetuin-A in overweight subjects with T2DM[46].

As stated in Table 3 and Table 4, energy intake and body weight significantly reduced after two months of *L. casei* supplementation in subjects with T2DM. Considering the effect of calorie restriction and weight loss on SIRT1 and fetuin-A levels, it can be understood that by reducing the appetite and dietary intake and body weight, probiotics could affect the plasma level of SIRT1 and fetuin-A in patients with T2DM in present trial.

*L. casei* supplementation for eight weeks significantly affected dietary intake and anthropometric indexes, including weight, BMI, and waist circumference in patients with T2DM. The effect of probiotics on gut microbial composition can affect appetite and food intake and also body composition and weight[4,7]. By modulating the gut microbiota, probiotics can affect the energy balance and/or metabolism of the host. Limited evidence exists on the effect of probiotic consumption on weight management in humans. The findings of present trial were similar to the study performed by Kadooka *et al*.[49] in which they reported that a supplementation of fermented milk with *Lactobacillus gasseri* for three months induced a significant weight loss and a decrease in BMI, hip, and waist circumferences and body fat mass. Omar *et al*.[50] have suggested that the consumption of *Lactobacillus* decreases total body fat mass.

A possible way for manipulating the mammalian eating behavior and body weight by probiotic bacteria is appetite-regulating hormones. Supplementation with VSL#3, containing Lactobacillus strains, in mice reduced appetite-inducing hormones and neuropeptide Y in the hypothalamus[51,52]. Moreover, the levels of leptin, cholecystokinin, and other satiety peptides, regulating appetite and food intake through affecting vagus nerve signaling, were improved[53]. Probiotics can control metabolism and energy intake by the production of SCFAs (short chain fatty acids) through fermentation of indigestible polysaccharides[7]. SCFAs, produced by bacterial fermentation, can regulate satiety and food intake[54]. Through activating the G protein-coupled receptors (GPR41 nd GPR43) on intestinal epithelial cells, SCFAs stimulate GLP1 and peptide YY secretion[7]. Based on the results of present survey, probiotics affected patients’ weight, BMI, and waist circumference by influencing dietary intake.

Taken together, this study demonstrates that *L. casei* could improve glycemic response and SIRT1 and fetuin-A levels. Taking into account the metabolic impacts of SIRT1 and fetuin-A, management of their levels could be effective in diabetes control. The results of present trial help us to reveal a new mechanism of probiotics action in diabetes and its related metabolic disorders control. Besides, as shown in this study, the positive effects of probiotics on body weight could be translated into favorable metabolic effects and have beneficial effects on the homeostasis of glucose.
